# Reproducibility of a novel echocardiographic 3D automated software for the assessment of mitral valve anatomy

**DOI:** 10.1186/s12947-016-0061-8

**Published:** 2016-05-17

**Authors:** Iolanda Aquila, Ariana González, Covadonga Fernández-Golfín, Luis Miguel Rincón, Eduardo Casas, Ana García, Rocio Hinojar, José Julio Jiménez-Nacher, José Luis Zamorano

**Affiliations:** 1 Cardiac Imaging Unit, Ramón y Cajal University Hospital, Carretera de Colmenar Km 9,100, 28034 Madrid, Spain; 2 Cardiovascular Institute, Department of Medical and Surgical Sciences, Magna Graecia University, Campus S. Venuta, Viale Europa, Catanzaro, 88100 Italy

**Keywords:** Mitral valve, 3D echocardiography, Automatic software

## Abstract

**Background:**

3D transesophageal echocardiography (TEE) is superior to 2D TEE in quantitative anatomic evaluation of the mitral valve (MV) but it shows limitations regarding automatic quantification. Here, we tested the inter-/intra-observer reproducibility of a novel full-automated software in the evaluation of MV anatomy compared to manual 3D assessment.

**Methods:**

Thirty-six out of 61 screened patients referred to our Cardiac Imaging Unit for TEE were retrospectively included. 3D TEE analysis was performed both manually and with the automated software by two independent operators. Mitral annular area, intercommissural distance, anterior leaflet length and posterior leaflet length were assessed.

**Results:**

A significant correlation between both methods was found for all variables: intercommissural diameter (*r* = 0.84, *p* < 0.01), mitral annular area (*r* = 0.94, *p* > 0, 01), anterior leaflet length (*r* = 0.83, *p* < 0.01) and posterior leaflet length (*r* = 0.67, *p* < 0.01). Interobserver variability assessed by the intraclass correlation coefficient was superior for the automatic software: intercommisural distance 0.997 vs. 0.76; mitral annular area 0.957 vs. 0.858; anterior leaflet length 0.963 vs. 0.734 and posterior leaflet length 0.936 vs. 0.838. Intraobserver variability was good for both methods with a better level of agreement with the automatic software.

**Conclusions:**

The novel 3D automated software is reproducible in MV anatomy assessment. The incorporation of this new tool in clinical MV assessment may improve patient selection and outcomes for MV interventions as well as patient diagnosis and prognosis stratification. Yet, high-quality 3D images are indispensable.

## Background

The prevalence of severe valvular diseases increases with age [1] thus representing an important public-health problem. In Europe, mitral regurgitation is the second most frequent valve disease requiring surgery [2]. Non-invasive mitral valve (MV) anatomical and functional evaluation is essential to define patient’s management. Despite the increasing number and availability of alternative imaging modalities, echocardiography remains the cornerstone in the assessment of MV morphology and physiology [3].

Over the last 5 decades, Echocardiography has evolved from mono-dimensional and two-dimensional (2D) imaging to sophisticated 3-dimensional (3D) techniques, introducing a new era for cardiovascular imaging [4]. 2D echocardiographic transesophageal approach increases diagnostic accuracy; however, the complex anatomy of the so-called MV apparatus remains in many cases still a challenge. 3D transesophageal echocardiography (TEE) has proved to overcome some of the 2D echocardiographic limitations in MV assessment, providing more accurate geometric information of the MV than 2D TEE [5, 6]. Several studies have shown the superiority of 3D TEE in the evaluation of the MV normal and pathologic morphology, quantification of mitral regurgitation or stenosis [7–12] and comprehensive evaluation of MV prolapse before surgery [13, 14].

However, 3D echocardiography has specific limitations. It requires training for both image acquisition and analysis. The image post-processing is time consuming, with low inter- and intra-observer reproducibility of manual measurements in many cases. The latter has created the need to develop automatic software able to both reduce image analysis time and increase reproducibility. Available computational geometric and biomechanical software require the user’s identification of MV structures as well as manual tracing, being time consuming and limiting reproducibility [15–18].

New automatic software for MV analysis as the one evaluated in the present study are promising for its use in clinical practice. Reproducibility is key point for the quantitative evaluation of non-invasive imaging techniques. Moreover, they constitute one of the main limitations of conventional echocardiography affecting patient’s diagnosis and management. For this reason new technological developments, need to prove their efficacy with higher reproducibility before they can be used in clinical practice replacing the available conventional methods. Accordingly, the aim of our study was to evaluate inter- and intra-observer reproducibility of a novel full-automated software in the evaluation of MV anatomy compared to routine clinical manual 3D assessment.

## Methods

### Patients

A total of 331 patients referred to the Cardiac Imaging Unit for TEE from January to September 2013 were initially screened for this study. Of the total screened population, 88 patients underwent TEE using an echocardiographic system not compatible with the automated software analysis. Of the remaining 243 patients with a TEE preformed with an iE33 ultrasound imaging system (Philips Medical System, Andover, MA), 80 patients had a 3D zoom of the mitral valve (MV). Of these 80 patients, 19 patients were excluded because they had a mitral prosthesis implant. Of the remaining 61 patients, 25 patients were excluded because of (i) poor-quality 3D images with stitching artefacts due to arrhythmia, (ii) a frame rate below 7 volumes per second or (iii) poor image quality for the software automatic quantification (i.e., incomplete imaging of the mitral annulus). Thus, final study population retrospectively included a total of 36 patients with 15 patients in sinus rhythm, 16 patients in atrial fibrillation and 5 with PM-dependent rhythm.

### Echocardiography

All patients underwent TEE according to the European Association of Cardiovascular Imaging Guidelines [19] using a multiplane transoesophageal 7X-2 t matrix probe. Both clinical TEE examination and 3D MV images were undertaken according to the performing physician. 3D MV images were obtained using 3D zoom modalities acquired over one cardiac cycle with frame rate ranging from 7 to 34 volumes per second except for two patients for which the images were obtained over 4 cardiac cycles. Images were digitally stored and transferred to a workstation for offline analysis.

### 3D data analysis

**Fig. 1:**
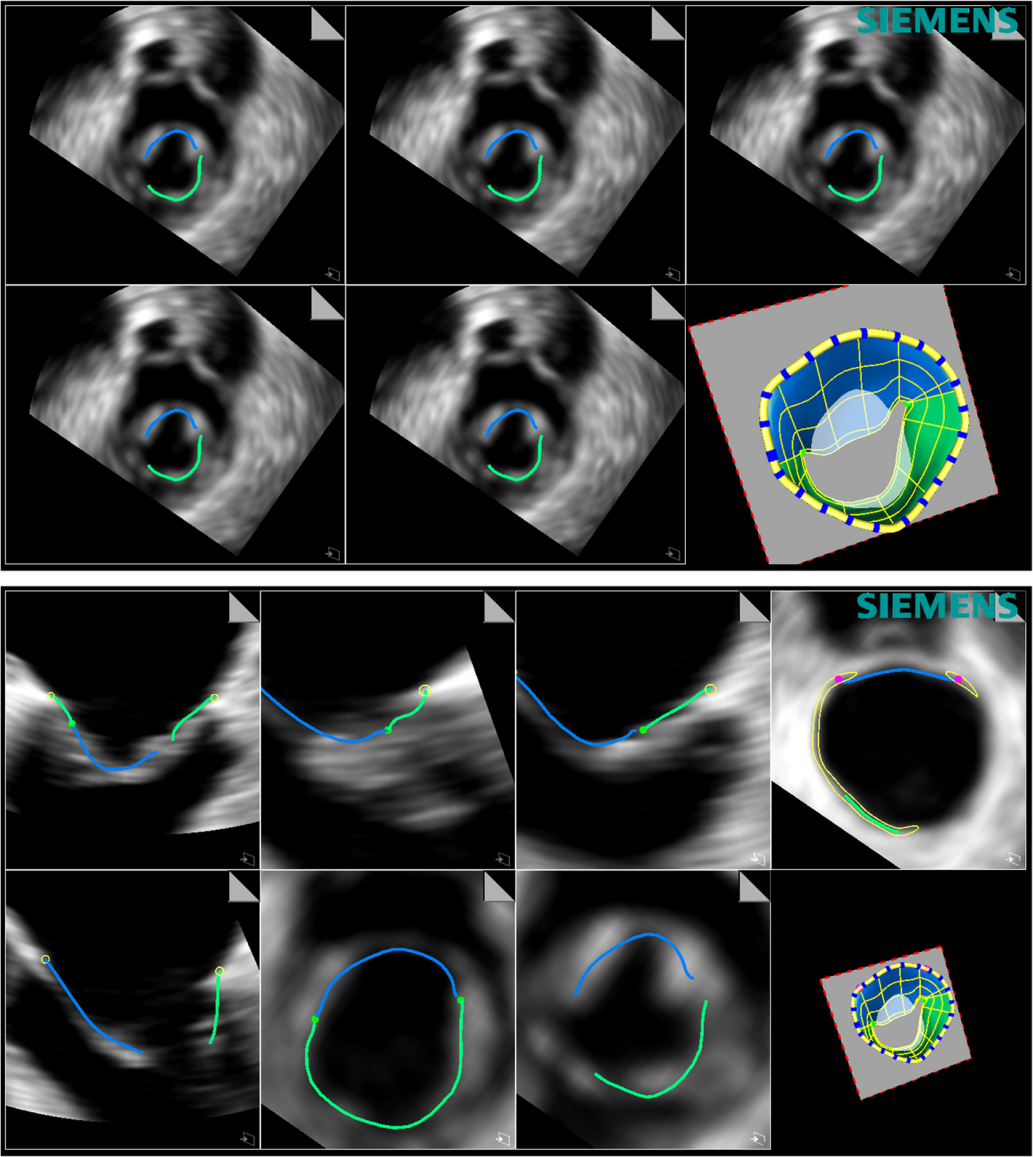
Representative image of the automatic software analysis of the mitral valve. Different views of the mitral valve are shown with the different structures automatically detected and the final 3D modelling of the mitral valve

**Fig. 2:**
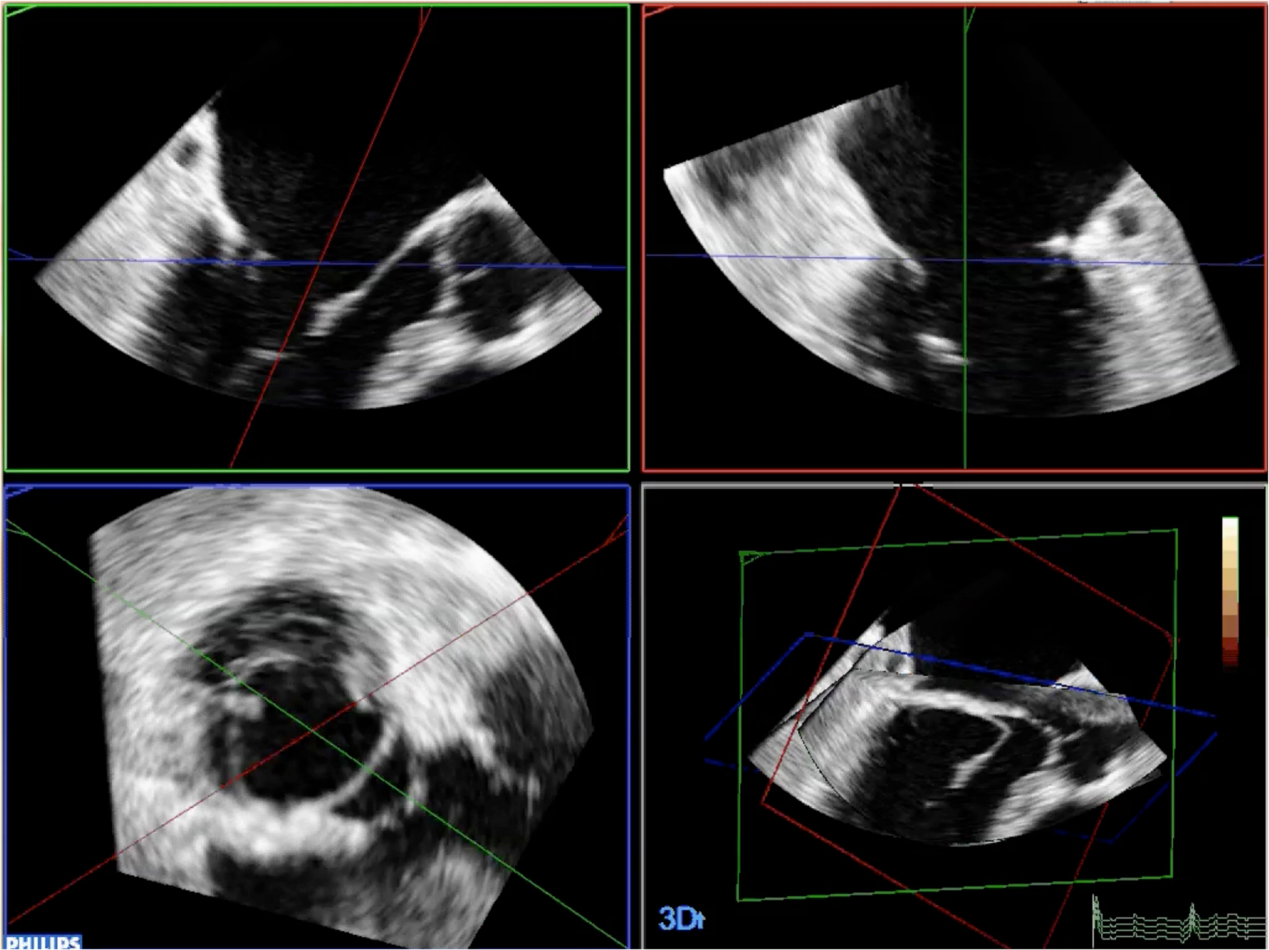
Representative image of a multiplanar reconstruction of the 3D dataset as obtained by manual measurements of the MV

In each subject the highest quality 3D images were selected for analysis. Same volume dataset and frame were used for both manual and software analysis, which included the following parameters: intercommissural distance, the area of mitral annulus and the leaflets length. After importing the images into the software (eSie Valves, Autovalve prototype version 1.22, Siemens Medical Solutions, USA) the MV is shown in different views (Fig. 1). It is worth noting here that currently Philips and Siemens are the echocardiographic system compatible with the automated software analysis. Following manual selection of the appropriate frame, automatic recognition of the MV is performed by the detection of 7 landmarks and more than 400 additional mitral annulus landmarks obtained in 50 different planes [15] that can be edited as needed. A MV model is computed based on these landmarks and different MV parameters are obtained. Manual measurements of the MV were performed using QLab 11; Philips Medical System. Multiplanar reconstruction of the 3D dataset was performed. Orthogonal axis was aligned obtaining 3 different MV planes: 4 chambers, 3 chambers and short axis view. The MV parameters were assessed at the time of maximal valve opening (mid diastolic frame). Both planimetry of the MV annulus and intercommissural diameter were assessed in the short axis view. Subsequently the leaflets length was measured in mid to late diastolic time in the 3 chambers view (Fig. 2).

### Statistical analysis

All quantitative data obtained by manual and software assessments were presented as mean ± standard deviation (SD). Comparison between both techniques was performed using a paired Student’s t-test. Differences were considered significant at *p* <0.05, and linear regression analysis was used to test correlation of variables. To test inter-observer variability between both methods, all images were analysed by 2 cardiologists that independently reviewed the 3D images and made both the manual measurements and used the automated software. One observer repeated the measurements in 15 randomly selected cases at 2 different time points to assess intra-observer variability. The inter-observer and intra-observer variability were analysed using the Bland-Altman method [20]. Inter-observer and intra-observer agreements for qualitative analysis score by 3D Echo manual and software assessments were calculated using intraclass correlation coefficients and classified as excellent with value of 0.93–1.0, very good 0.81–0.92, good 0.41–0.60, and poor < 0.4 classified as low if ICC < 0.4, fair if ICC 0.4–0.75 and excellent if ICC > 0.75 [21]. Statistical analyses were performed using SPSS version 22.0 (SPSS, Inc., Chicago, Illinois) and Stata SE version12.0 (StataCorp, Texas).

## Results

Clinical characteristics of the study cohort are shown in Table 1. 6 (17 %) patients had normal mitral valve, 9 (25 %) mitral regurgitation due to isolated MV prolapse or associated to chordae tendinae rupture, 9 (25 %) degenerative MV disease, 8 (22 %) rheumatic valve disease, 3 (8 %) functional mitral regurgitation and 1 (3 %) patient had MV perforation secondary to endocarditis.

**Table 1:** Baseline characteristics of the study population

	*n* (%)
Age (years)	72 ± 12,4
Gender, male n (%)	17 (47 %)
Hypertension, n (%)	28 (78 %)
Hypercholesterolemia, n (%)	11 (31 %)
Diabetes mellitus, n (%)	11 (31 %)
Smoking, n (%)	9 (25 %)
Sinus rhythm, n (%)	15 (42 %)
Atrial fibrillation, n (%)	16 (44 %)
Ejection fraction (EF) < 45 %.	4 (11 %)

**Fig. 3:**
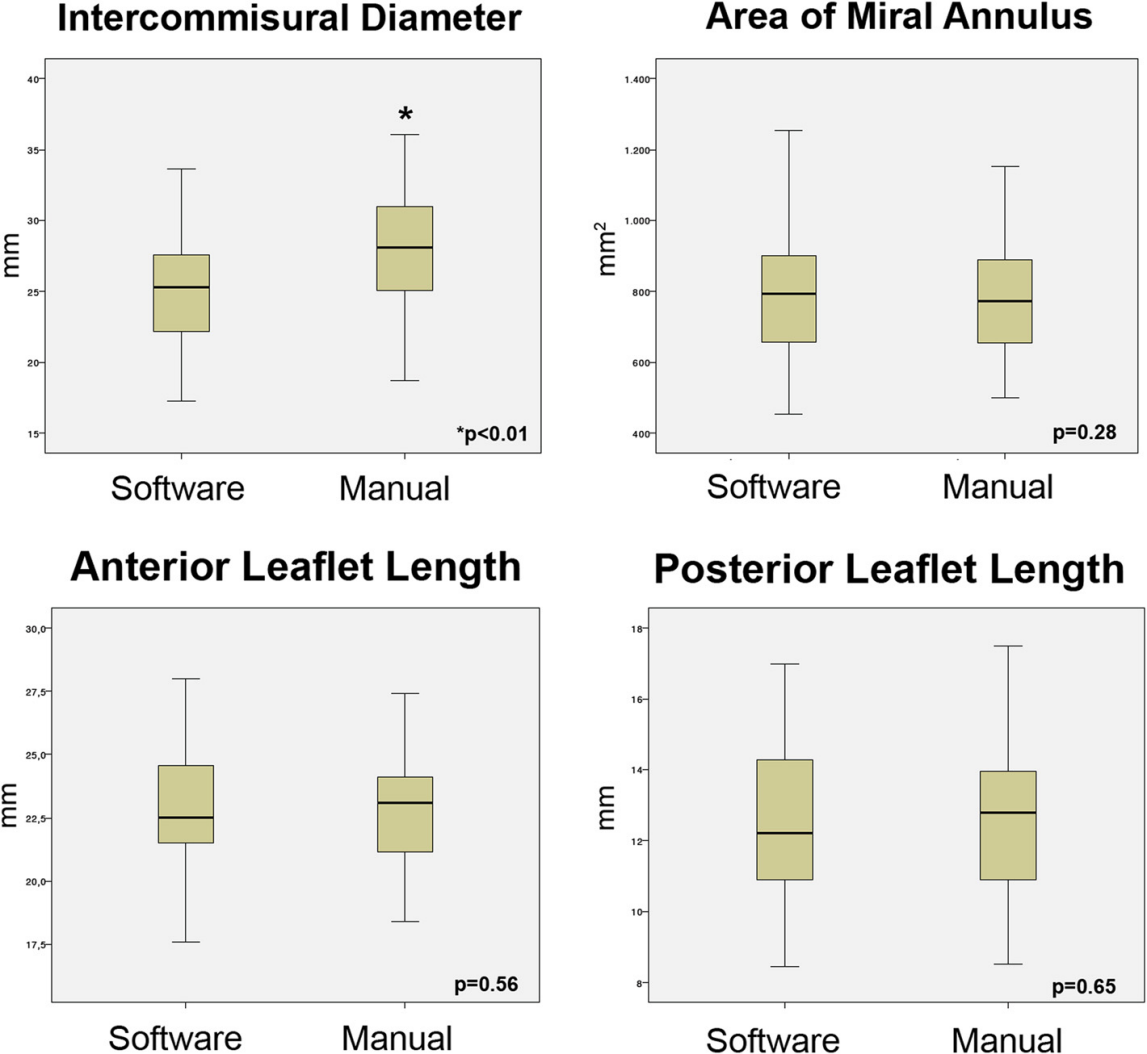
Box plots with the cumulative data of the four mitral valve anatomical parameters as assessed by the automatic software versus manual evaluation

**Fig. 4:**
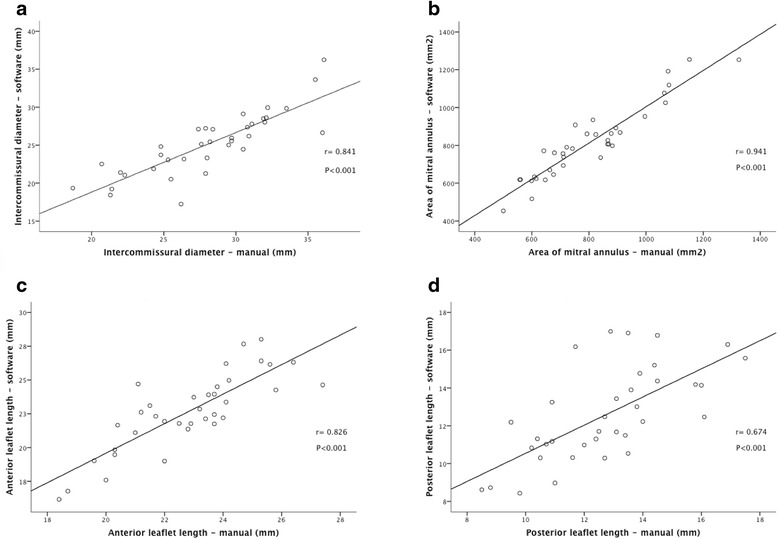
Correlations between intercommissural diameter (**a**), area of mitral annulus (**b**), anterior leaflet length (**c**) and posterior leaflet length (**d**) determined by automated software and manual assessment

**Fig. 5:**
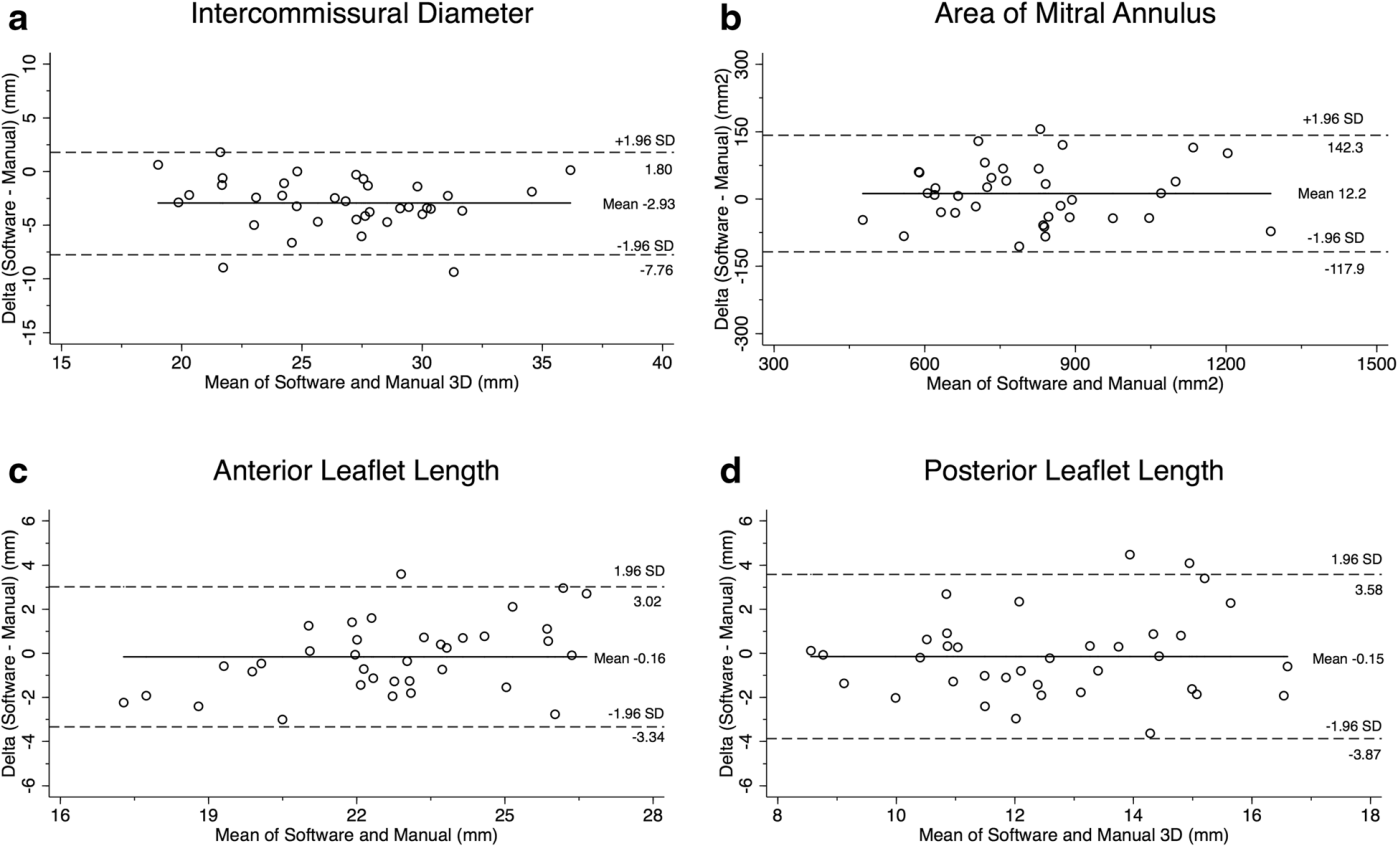
Bland-Altman scatterplot demonstrating the agreement in the measurement of MV intercommissural diameter (**a**), area of mitral annulus (**b**), anterior leaflet length (**c**) and posterior leaflet length (**d**) measured by automated software and manual assessment. Intercommissural diameter measured using automated software was underestimated when compared to manual evaluation. The *solid horizontal line* in each plot represents the mean systematic difference (bias) between the two methods, whereas the *dashed lines* indicate the limits of agreement (95 % confidence interval of differences)

No significant differences were noted between all parameters but intercommissural distance (Table 2, Fig. 3). Manual assessment of intercommissural diameter was significantly correlated with intercommissural diameter by the automated software (*r* = 0.84, *p* < 0.01; Fig. 4), but manual calculation resulted in higher measurements compared to those measured using automated software (mean difference -2.93 ± 2.41; *p* < 0.01) (Fig. 5). Area of mitral annulus and anterior valve length assessed manually and with the automated software show strong correlations (*r* = 0.94 and *r* = 0.83 respectively, all *p* < 0.01) and good levels of agreement (Figs. 4 and 5). Correlation of posterior leaflet length by manual assessment and automated software was less strong (*r* = 0.67, *p* < 0.01) but did not show significant differences regardless of the method used (mean difference −0.15 ± 1.9, p 0.65) (Figs. 4 and 5).

**Table 2:** Mitral valve anatomical parameters

	3D manual assessment (*n* = 36)	3D automated software (*n* = 36)	*p* value*
Intercommissural diameter (mm)	28.1 ± 4.4	25.16 ± 4.1	0.00
Area of mitral annulus (mm^2^)	802.5 ± 190.8	814.7 ± 194.4	0.28
Anterior leaflet length (mm)	22.8 ± 2.2	22.7 ± 2.9	0.56
Posterior leaflet length (mm)	12.7 ± 2.2	12.6 ± 2.5	0.65

*Automated software versus manual assessment paired Student t-test

3D automated Software measurements showed a better interobserver agreement in all the imaging parameters. Intraclass correlation coefficient for 3D manual and 3D automated Software measurements respectively was 0.76 (95 % CI 0.58 to 0.87) and 0.99 (95 % CI 0.99 to 0.99) for intercommissural distance; 0.85 (95 % CI 0.73 to 0.92) and 0.95 (95 % CI 0.91 to 0.97) for area of mitral annulus; 0.734 (95 % CI 0.53 to 0.85) and 0.963 (95 % CI 0.92 to 0.98) for anterior leaflet length; 0.838 (95 % CI 0.705 to 0.941) and 0.936 (95 % CI 0.879 to 0.967) for posterior leaflet length.

Intraobserver variability showed a superior agreement with 3D automated Software for all measurements but posterior leaflet length. Intra-class correlation coefficient was: intercommissural distance, 0.938 [95 % confidence interval (CI) 0.827 to 0.979] for 3D manual assessment and 0.999 (95 % CI 0.998 to 1.0) for the 3D automated Software; area of mitral annulus, 0.969 (95 % CI 0.991 to 0.990) for 3D manual assessment and 0.992 (95 % CI 0.975 to 0.997) for the 3D automated software; anterior leaflet length, 0.882 (95 % CI 0.685 to 0.959) for 3D manual assessment and 0.984 (95 % CI 0.954 to 0.995) for the 3D automated Software; posterior leaflet length, 0.946 (95 % CI 0.846 to 0.981) for 3D manual assessment and 0.937 (95 % CI 0.824 to 0.978) for the 3D automated Software.

## Discussion

The main findings of the present study are that: 1) the 3D automated software correctly evaluates MV morphology and dimensions; 2) the MV evaluation by the automated software strongly correlates with the operator´s manual analysis; 3) the software analysis is reliable, reproducible and operator-independent.

MV disease prevalence is high, with a growing number of patients requiring intervention. The increasing number and complexity of both surgical and percutaneous treatments, with clear anatomic requirements (edge-to-edge MV repair technique using MitraClip) demands imaging techniques to be more precise, accurate and reproducible for both patient selection and intraprocedural control [22]. Although 3D TEE offers a better diagnostic accuracy compared to 2D TEE, it has not yet become widespread in the clinical routine. Commercially available 3D echocardiography analysis packages allow only for a limited number of quantitative measures to be performed offline. Custom software algorithms that allow interactive visualization and automated quantification have been developed, but these techniques are time consuming, and labour intensive with poor reproducibility [15]. For all the above reasons, the increased interest in a precise morphological and functional evaluation of the MV has reinforced the need to introduce into clinical practice 3D models able to generate a detailed morphological reconstruction as well as sophisticated quantification of the complex MV structure and dynamics throughout the cardiac cycle with high precision [23].

To the best of our knowledge, no clinical studies have assessed the feasibility and reproducibility of quantification of the MV complex using the automated software eSie Valves as shown in the present study. Our results show no significant differences between mean values obtained with the software and 3D manual assessment except for the intercommissural distance (Table 2, Fig. 3). In this parameter, even though Pearson correlation coefficient was high (Fig. 4), the software systematically showed lower results (Fig. 5). This is easily explained by the different methodology used in both methods: commissures are detected by the software in the 3D space and then the Euclidian distance between them is calculated. However, in the 3D manual analysis, the commissures are identified in 2D images after multiplanar reconstruction, in the short axis view and a straight line is traced between them. Moreover, commissures are not exact anatomical landmarks, and represent the continuity between the anterior and posterior leaflet extending over some millimetres. Additionally, intercommissural distance is not the main and standard parameter to guide prosthesis size and thus it should not impact the use of the automated software for percutaneous mitral prosthesis implantation in the future. A good correlation was found between the rest of the measurements performed with the software and manual 3D analysis (Fig. 4) without significant bias (Fig. 5). The lowest correlation was seen for the posterior mitral leaflet length but no significant differences in mean values were seen whatsoever. This may be due to the fact that posterior leaflet length was manually measured at P2, while the software computed maximal posterior leaflet length, which in some cases may be a little different. However, grade of agreement did not show significant bias either. Additional MV parameters obtained with the software were not included in the analysis. This was decided due to inherent limitations of the 3D manual assessment that would have limited the evaluation of software´s accuracy. Since there is no clear gold standard for most of MV parameters obtained, clinical studies are needed in order to prove their accuracy and usefulness. Despite it was not specifically addressed, the automatic software reduced of at least half the time for analysis when compared to manual evaluation further underlying the clinical usefulness of the software. However, further studies are needed to specifically quantify the time-saving by the automatic software.

Regarding inter and intraobserver reproducibility, our work confirms superiority of the automated software over 3D manual assessment. These findings are in agreement with a previous report that tested reproducibility of the same software in 18 patients undergoing coronary bypass surgery [23].

The increasing number of interventional procedures demands non-invasive imaging techniques to be more reproducible for its use in clinical practice. Those not able to fulfil these requirements will be left behind in the near future where new surgical and interventional devices develop to treat different forms of MV disease. In this regard, the future of 3D echocardiography requires not only to generate superb image quality with high temporal and spatial resolution but also to offer reproducible quantification and to be operator independent. For these reason results of the present study are of paramount importance, highlighting the ability of technical developments, as the software evaluated, to respond to real needs of echocardiography. Reproducibility results obtained in two separate studies further reinforce its strengths and support its applicability in every day clinical practice.

### Limitations

First, we did not compare our measurements performed manually and by the software with pathologic findings during cardiac surgery in absence of a gold standard able to define a correct validation of the measurements made. Second, in 41 % of patients it was not possible to perform the analysis retrospectively. This translates the importance of high quality 3D images and the need to include the entire MV annulus in the acquired volume. Third, the study patients’ cohort is clearly inhomogeneous as it spans from normal mitral valve to different aetiologies of mitral valve disease. However, it should be pointed out that in this study we specifically assessed the reproducibility of the automated software analysis compared to manual evaluation of the mitral valve apparatus independently from the presence or absence of any pathology of the valve. Finally, the automated software analysis includes by default of a number of other parameters (like mitral orifice area, tenting volume, tenting height, annulus non planarity angle scalar etc. etc.) that were not reported in this study. This is so because these additional parameters are not part of a standard manual evaluation and thus they were not used for the comparison.

## Conclusion

The new eSieValves software has proved to be reproducible in MV anatomic evaluation. From high quality 3D TEE images, it allows the possibility of correctly analysing several MV parameters in one frame and over the cardiac cycle, opening new possibilities in the understanding of physiology and pathology of this complex structure. These features may improve not only surgical and interventional procedures planning but also diagnosis and prognosis stratification in MV disease patients. Further clinical studies are needed to define clinical application of the MV parameters obtained.
